# Supplementary material for: Identification of the suprachiasmatic nucleus venous portal system in the mammalian brain
Source: Nat Commun. 2021 Sep 24;12:5643. doi: 10.1038/s41467-021-25793-z (PMC8463669; doi:10.1038/s41467-021-25793-z)
Supplement: Supplementary file 1 — Supplementary Information [file 41467_2021_25793_MOESM1_ESM.pdf]

Supplementary information for

The portal system of the suprachiasmatic nucleus

Yifan Yao[1], Alana Taub[1], Joseph LeSauter[2], Rae Silver* [1,2,3]

1 Columbia University Department of Psychology, 1190 Amsterdam Avenue, New York City, New York, 10027, USA.

2 Department of Neuroscience, Barnard College, 3009 Broadway, New York City, New York, 10027, USA.

3 Department of Pathology and Cell Biology, Graduate School, Columbia University Medical School, New York City, New York, 10032, USA.

*Correspondence should be addressed to R.S (Rae.Silver@columbia.edu)

Supplementary figures and figure legends

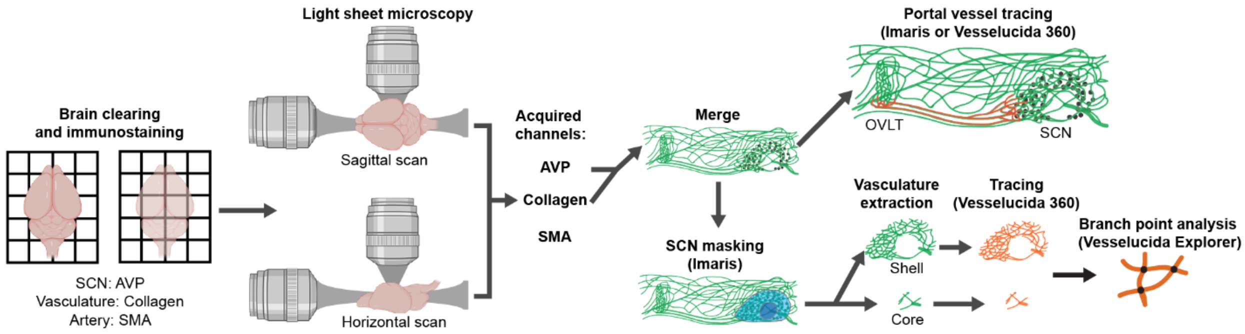

Supplementary Fig. 1 Experimental pipeline. The cleared brains were labelled with AVP to localize the SCN, collagen to label the entire vasculature, and SMA to label the arteries. The samples were scanned either sagittally or horizontally. The collagen channel was used for blood vessel tracing, and the boundaries of shell and core SCN were drawn based on the AVP staining. The vasculature of the core and shell SCN was extracted and traced separately and the traces were exported for branch point analysis. Created with BioRender (https://biorender.com/).

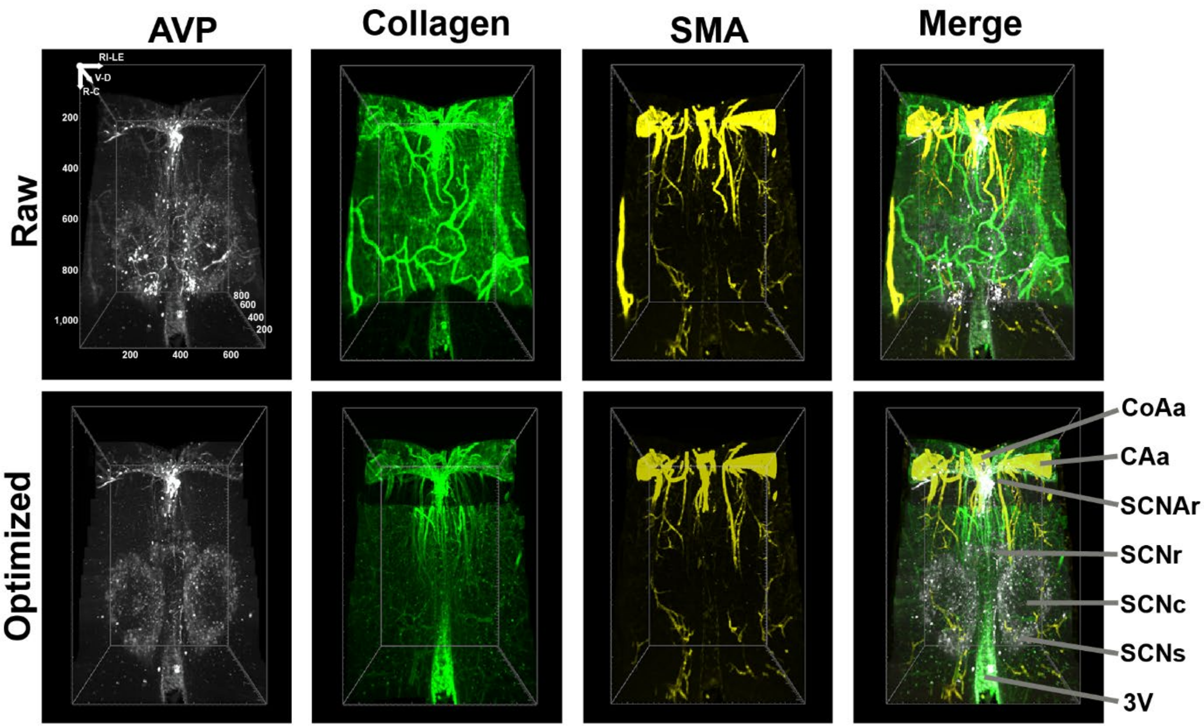

Supplementary Fig. 2 Horizontal view of portal vasculature between the SCN and OVLT. AVP, collagen, SMA and the merged channels are shown in both rows. In the "optimized" bottom row, masking was used to enable simultaneous visualization of very intense (collagen in OVLT) and less intense (collagen in SCN) and to eliminate background signal from the pia and optic chiasm. AVP demarcates the SCN main body and the rostrum which lies in the midline and forms the rostral projection of the nucleus and the bridge that joins the two sides. In the collagen channel, a rich bundle of blood vessels lies along the midline. SMA labels arteries. The merged image shows the midline blood vessels that travel between the SCN and OVLT. Arteries branching from the CAa and CoAa, which supply the SCN, are lateral to the midline blood vessels. Abbreviations: CoAa=anterior communicating artery; SCNAr=SCN artery, rostral branch; remaining abbreviations as in Fig. 1&3.

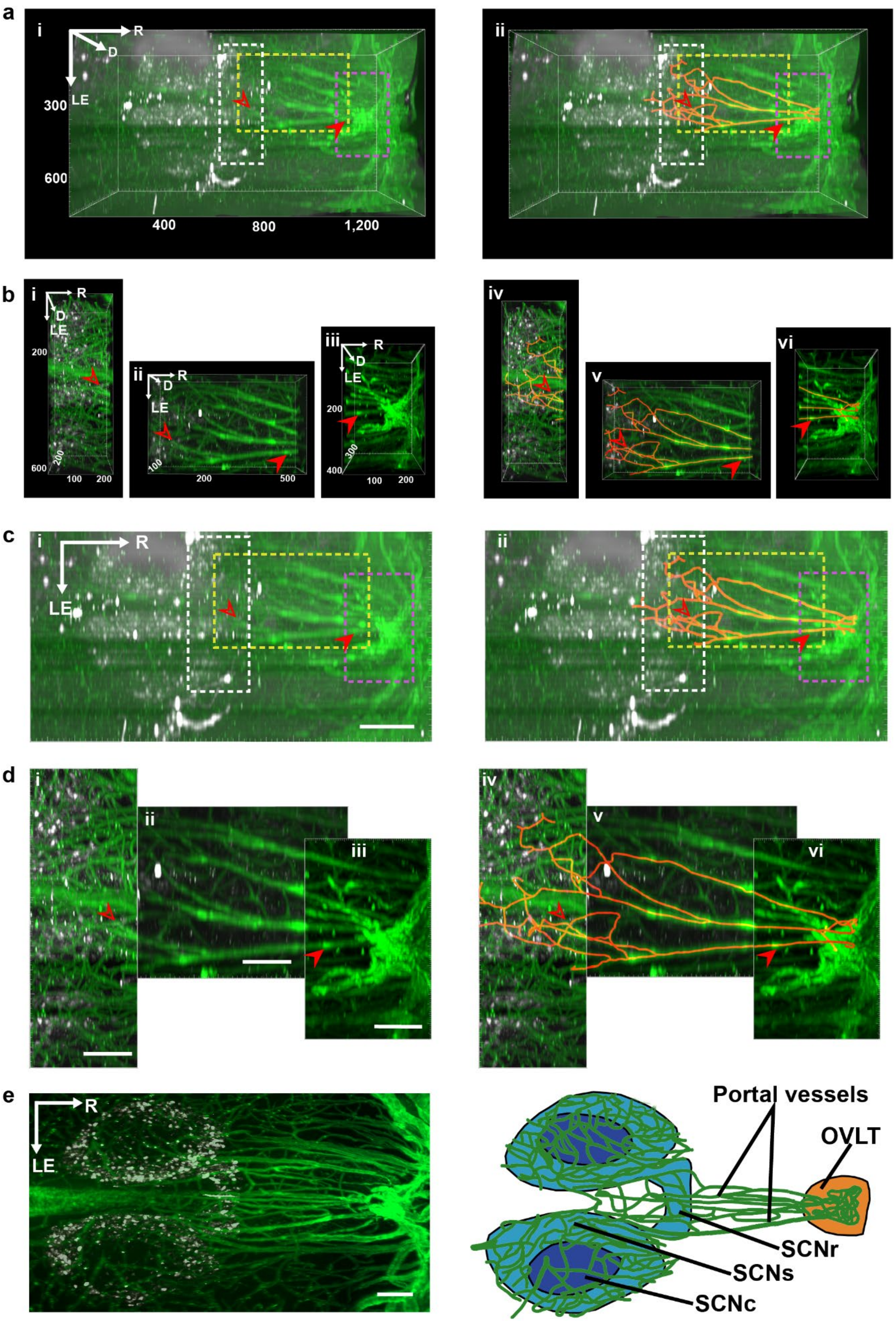
a
i
R
D
LE
300
600
400
800
1,200
ii
b
i
R
D
LE
200
600
200
100
200
ii
R
D
LE
100
200
500
iii
R
D
LE
200
300
400
100
200
iv
v
vi
c
i
R
LE
ii
d
i
ii
iii
iv
v
vi
e
R
LE
Portal vessels
OVLT
SCNr
SCNs
SCNc

Supplementary Fig. 3 Horizontal view of portal vasculature between the SCN and OVLT in 3D and maximum intensity projection views. a-d The left panels show capillaries coursing between the SCN and OVLT and the right panels show the same panels with traced vessels. **a, b** 3D view of vessels between SCN and OVLT at low (a) and higher (b) magnification. Regions outlined in the boxes in (a) are shown at higher magnification for the region (left to right) near the SCN**,** between SCN and OVLT**,** and near OVLT. **c, d** Maximum intensity projection of data shown in a, b respectively at low (c) and high magnification (d). This view reduces the depth of the scan and enhances visualization of some of the portal vessels coursing between the SCN and OVLT (N=8 mice). **e** Maximum intensity projection of an optical slice (left, depth=100µm) and accompanying schematic (right) highlight the entry points of portal vessels into the ventral superficial plexus of the OVLT (N=8 mice). Legend details: In panels a-d, the open and closed arrows point to a specific vessel lying at the rostral SCN or near the ventral OVLT respectively. Immunostaining labels as in Fig 2 and orange=tracings of capillaries. For a-b volume of images is shown in the axes. For panels c-d, maximum intensity projections depth: 620µm (i, iv), 350µm (ii, v), 410µm (iii, vi). Reference axes as in Fig. 2. Scale bar=100µm.

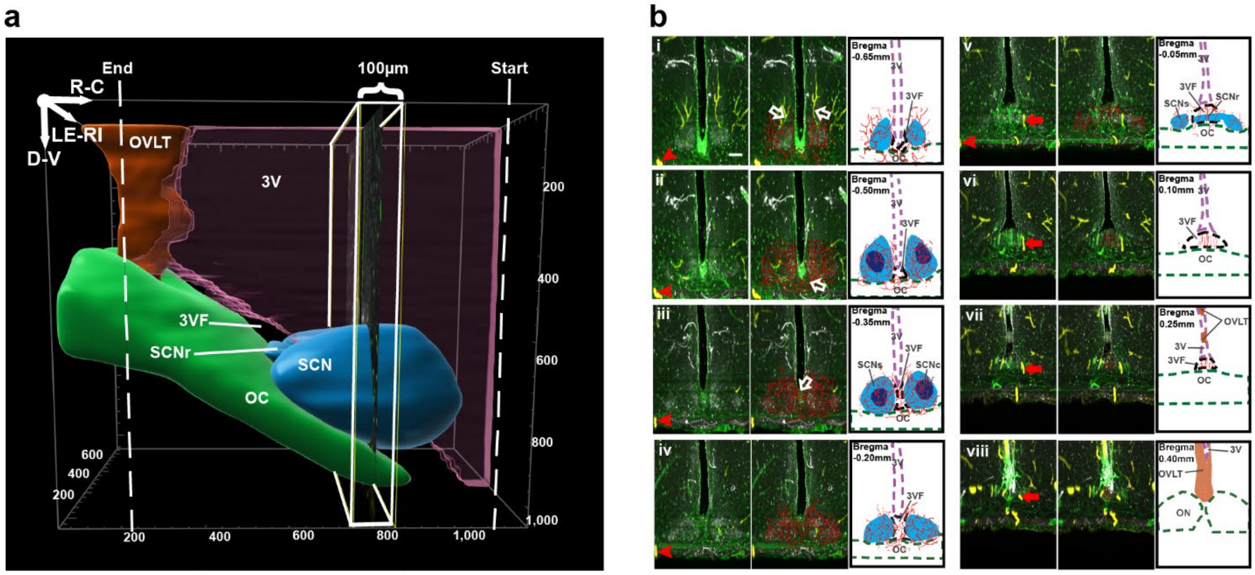

Supplementary Fig. 4 Coronal view of capillaries connecting SCN and OVLT. a The white rectangle shows the orientation of the coronal serial sections made from the SCN to the OVLT starting from the caudal aspect of the SCN. **b** Serial optic slices (50µm) of vasculature between SCN and OVLT from Bregma -0.65 to 0.40 demonstrating continuity of portal capillaries (N=6 mice). The plates show triplets of images as in Fig. 3. Details of the serial plates are as follows: **bi,** In the caudal-most SCN, arteries run vertically through the nucleus (middle column, white arrows). **bii-iv** The SCN vasculature between hemispheres are connected by capillaries lying in the chiasm (bii**,** middle column, white arrow) and in the 3VF (biii, middle column, white arrow). **bv** Blood vessels from bilateral SCNs form anastomoses near SCNr. **bvi-vii** Portal blood vessels travel in the 3VF. **bviii** Portal blood vessels join OVLT capillary network at its base. Abbreviations and colour codes as in Fig. 3. Scale bar=100µm.

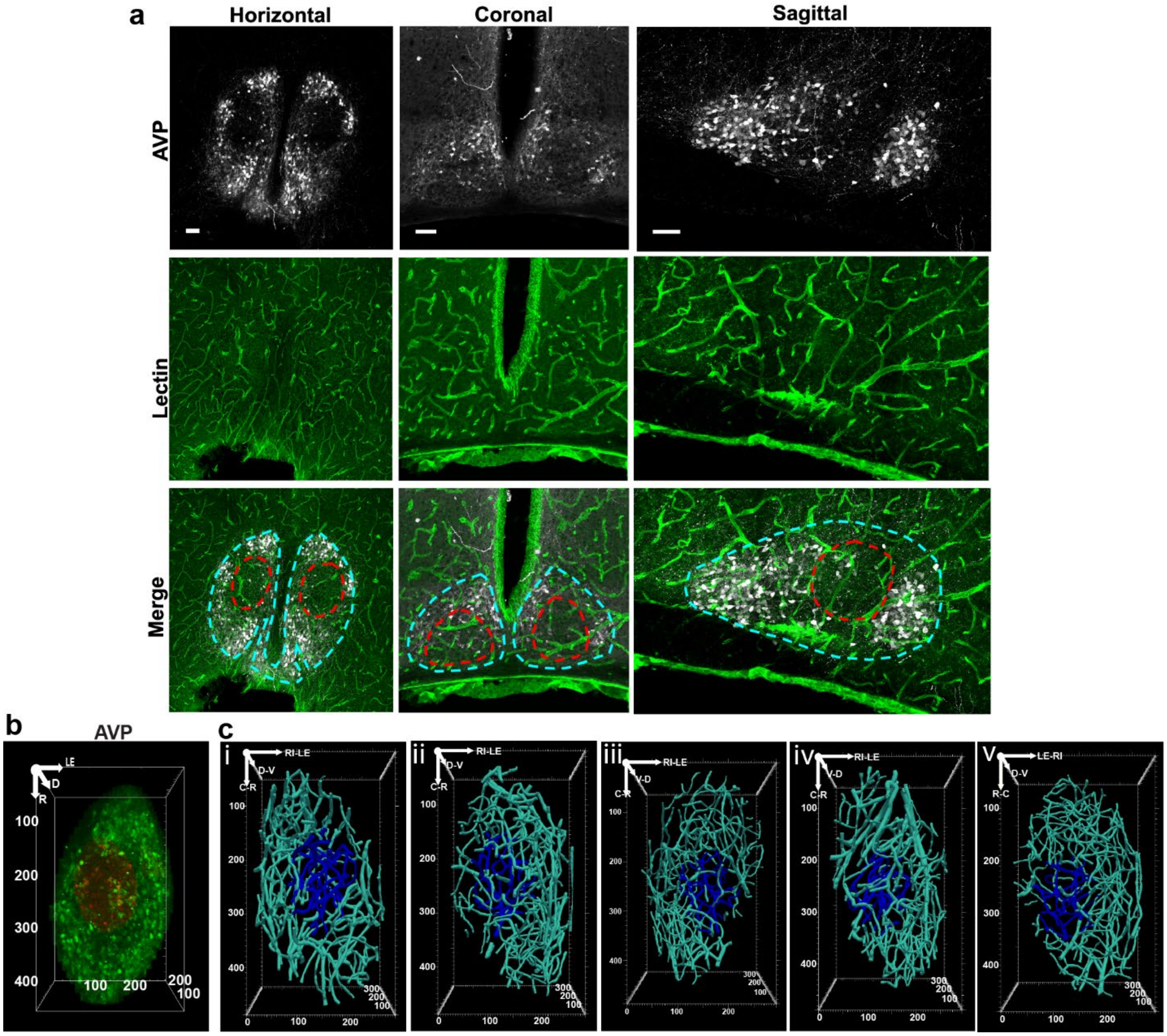

Supplementary Fig. 5 SCN shell vs. core. a Confocal microscopic images of SCN in three orientations distinguish the AVP poor core from the AVP rich shell (N=3 mice). Merged images, in each orientation, suggest that there is greater vasculature density in the SCN shell (dashed blue line) compared to the core (dashed red line). Best seen in the AVP-labelled horizontal view is the SCNr which lies right at the midline, the locus of capillary blood vessels connecting SCN and OVLT. Scale bar=50µm. **b** AVP staining in iDISCO cleared SCN after masking. SCN shell=green; SCN core=red. Reference axis: R=rostral; LE=left; D=dorsal. **c** The statistical

analysis in Fig. 4biii was based on traces of six SCNs (N=5 in Supplementary Fig. 5ci-v and N=1 in Fig. 4bii). Colour coding as in Fig. 4b.
