# Supplementary material for: Identification of the suprachiasmatic nucleus venous portal system in the mammalian brain
Source: Nat Commun. 2021 Sep 24;12:5643. doi: 10.1038/s41467-021-25793-z (PMC8463669; doi:10.1038/s41467-021-25793-z)
Supplement: Supplementary file 3 — Description of Additional Supplementary Files [file 41467_2021_25793_MOESM3_ESM.pdf]

Description of Additional Supplementary Files

File Name: Supplementary Movie 1
Description: 3D visualization of the SCN – OVLT portal system in the sagittal orientation. SCN labelled with AVP, white; vasculature identified with type IV collagen, green; traces of vascular connection between SCN and OVLT, orange.
