# Supplementary material for: Identification of the suprachiasmatic nucleus venous portal system in the mammalian brain
Source: Nat Commun. 2021 Sep 24;12:5643. doi: 10.1038/s41467-021-25793-z (PMC8463669; doi:10.1038/s41467-021-25793-z)
Supplement: Supplementary file 5 — Reporting Summary [file 41467_2021_25793_MOESM5_ESM.pdf]

nature portfolio

Corresponding author(s): Rae Silver

Last updated by author(s): Jul 28, 2021

Reporting Summary

Nature Portfolio wishes to improve the reproducibility of the work that we publish. This form provides structure for consistency and transparency in reporting. For further information on Nature Portfolio policies, see our Editorial Policies and the Editorial Policy Checklist.

Statistics

For all statistical analyses, confirm that the following items are present in the figure legend, table legend, main text, or Methods section.

n/a	Confirmed	
☐	☒	The exact sample size (*n*) for each experimental group/condition, given as a discrete number and unit of measurement
☐	☒	A statement on whether measurements were taken from distinct samples or whether the same sample was measured repeatedly
☐	☒	The statistical test(s) used AND whether they are one- or two-sided<br>*Only common tests should be described solely by name; describe more complex techniques in the Methods section.*
☒	☐	A description of all covariates tested
☐	☒	A description of any assumptions or corrections, such as tests of normality and adjustment for multiple comparisons
☐	☒	A full description of the statistical parameters including central tendency (e.g. means) or other basic estimates (e.g. regression coefficient) AND variation (e.g. standard deviation) or associated estimates of uncertainty (e.g. confidence intervals)
☐	☒	For null hypothesis testing, the test statistic (e.g. *F*, *t*, *r*) with confidence intervals, effect sizes, degrees of freedom and *P* value noted<br>*Give P values as exact values whenever suitable.*
☒	☐	For Bayesian analysis, information on the choice of priors and Markov chain Monte Carlo settings
☒	☐	For hierarchical and complex designs, identification of the appropriate level for tests and full reporting of outcomes
☒	☐	Estimates of effect sizes (e.g. Cohen's *d*, Pearson's *r*), indicating how they were calculated

Our web collection on statistics for biologists contains articles on many of the points above.

Software and code

Policy information about availability of computer code

Data collection	Light sheet microscopy images were acquired with lmSpector version 7.0.119 (LaVision BioTec)<br>Confocal images were acquired with NIS Elements Advanced Imaging Software version 5.10 (Nikon)
Data analysis	Image processing for confocal images:NIS Elements Advanced Research Imaging Software version 5.11 (Nikon)<br>Image processing for light sheet microscopy images:Imaris version 9.5.1 (Bitplane)<br>Tracing: Vesselucida 360 version 2019.1.3 (MBF Bioscience)<br>Vasculature network analysis: Vesselucida explorer 2019.1.1 (MBF Bioscience)<br>Statistics: R version 4.0.0 or higher (R Foundation for Statistical Computing)<br>Box-wisker plot: ggplot2 version 3.3.2 or higher (Wickham H (2016). ggplot2: Elegant Graphics for Data Analysis. Springer-Verlag New York. ISBN 978-3-319-24277-4, https://ggplot2.tidyverse.org.)

For manuscripts utilizing custom algorithms or software that are central to the research but not yet described in published literature, software must be made available to editors and reviewers. We strongly encourage code deposition in a community repository (e.g. GitHub). See the Nature Portfolio guidelines for submitting code & software for further information.

March 2021

Data

Policy information about availability of data

All manuscripts must include a data availability statement. This statement should provide the following information, where applicable:

- Accession codes, unique identifiers, or web links for publicly available datasets
- A description of any restrictions on data availability
- For clinical datasets or third party data, please ensure that the statement adheres to our policy

Data associated with this study are present in the paper or in the Supplementary Information file. The raw data that support the findings of this study are available from the corresponding authors upon reasonable request. Source data are provided with this paper.

Field-specific reporting

Please select the one below that is the best fit for your research. If you are not sure, read the appropriate sections before making your selection.

☒ Life sciences ☐ Behavioural & social sciences ☐ Ecological, evolutionary & environmental sciences

For a reference copy of the document with all sections, see nature.com/documents/nr-reporting-summary-flat.pdf

Life sciences study design

All studies must disclose on these points even when the disclosure is negative.

Sample size	We now report all N's. We used N=6 SCN to analyze the SCN core vs shell. We note that Kirst et al (Cell, 2020), a publication highlighted by a reviewer, analyzed branch point density studied N=3. For the iDISCO protocol, N=8 double labeled and N=6 triple labeled after optimization of immunostaining were used for examining SCN-OVLT vascular connections. Thirteen additional brains were uses in optimizing the iDISCO protocol. N=3 biologically independent samples were used for confocal microscopy.
Data exclusions	No data were excluded from the analysis
Replication	Double labeled iDISCO and light sheet imaging were done in 2 independent replicates and triple labeled iDISCO and light sheet imaging were done in 3 independent replicates. All attempts to replicate the data were successful.
Randomization	Of the 28 SCN's available, we randomly selected 6 SCN for branch density analysis. The comparison between the core and the shell is a within-subject design and randomization is not applicable.
Blinding	Tracing was done by 2 independent researchers and yielded the same results.

Reporting for specific materials, systems and methods

We require information from authors about some types of materials, experimental systems and methods used in many studies. Here, indicate whether each material, system or method listed is relevant to your study. If you are not sure if a list item applies to your research, read the appropriate section before selecting a response.

Materials & experimental systems

n/a	Involved in the study	
☐	☒	Antibodies
☒	☐	Eukaryotic cell lines
☒	☐	Palaeontology and archaeology
☐	☒	Animals and other organisms
☒	☐	Human research participants
☒	☐	Clinical data
☒	☐	Dual use research of concern

Methods

n/a	Involved in the study	
☒	☐	ChIP-seq
☒	☐	Flow cytometry
☒	☐	MRI-based neuroimaging

Antibodies

Antibodies used	Primary antibodies for iDISCO:<br>Anti-AVP (1:5000, rabbit, Immunostar, 20069, Lot#1911001)<br>Anti-Type IV collagen-UNLB (1:125, goat, SouthernBiotech, 1340-01, Lot#H2915-M709)<br>Anti-SMA (1:67, mouse, Clone 1A4, Dako, M0851, Lot#20067246)<br>Secondary antibodies for iDISCO:<br>Donkey anti-rabbit Cy2(1:200, Jackson ImmunoResearch, 711-225-152, Lot#146036)<br>Donkey anti-goat Cy5(1:200, Jackson ImmunoResearch, 705-175-147, Lot#144178)

	Donkey anti-mouse Cy3(1:200, Jackson ImmunoResearch, 715-165-151, Lot#145382)<br>Primary antibody for immunochemistry:<br>Anti-AVP antibody (1:5000, rabbit, Immunostar, 20069, Lot#1911001)<br>Secondary antibodies for immunochemistry:<br>Donkey anti-rabbit Cy3(1:200, Jackson ImmunoResearch, 711-165-152, Lot#144186)<br>Tomato Lectin, Fluorescien (1:100, Vector Laboratories, FL-1171, Lot#ZF0715)
Validation	All antibodies were commercial in origin. Validation statements can be found on the manufacturer's website as following.<br>Anti-AVP, https://www.immunostar.com/shop/antibody-catalog/vasopressin-antibody/<br>Anti-Type IV collagen-UNLB, https://www.southernbiotech.com/?catno=1360-01&type=Polyclonal#&panel2-1<br>Anti-SMA, https://www.agilent.com/en/product/immunohistochemistry/antibodies-controls/primary-antibodies/actin-(smooth-muscle)-(concentrate)-76542#references<br>Donkey anti-rabbit Cy2, https://www.jacksonimmuno.com/catalog/products/711-225-152<br>Donkey anti-goat Cy5, https://www.jacksonimmuno.com/catalog/products/705-175-147<br>Donkey anti-mouse Cy3, https://www.jacksonimmuno.com/catalog/products/715-165-151<br>Donkey anti-rabbit Cy3, https://www.jacksonimmuno.com/catalog/products/711-165-152<br>Tomato Lectin, Fluorescien, https://vectorlabs.com/fluorescein-labeled-lycopersicon-esculentum-tomato-lectin-lel-tl.html

Animals and other organisms

Policy information about studies involving animals; ARRIVE guidelines recommended for reporting animal research

Laboratory animals	10 -14 weeks old male C57BL/6NJ mice
Wild animals	This study didn't involve wild animals.
Field-collected samples	This study didn't involve samples collected from the field.
Ethics oversight	All experiments were performed according to protocols approved by the Institutional Animal Care and Use Committee of the Columbia University in accordance with guidelines set by the NIH (Protocol AC-AABH1603)

Note that full information on the approval of the study protocol must also be provided in the manuscript.
